# Emerging titanium surface modifications: The war against polymicrobial infections on dental implants

**DOI:** 10.1590/0103-6440202204860

**Published:** 2022-03-07

**Authors:** Valentim A. R. Barão, Raphael C. Costa, Jamil A. Shibli, Martinna Bertolini, João Gabriel S. Souza

**Affiliations:** 1 Department of Prosthodontics and Periodontology, Piracicaba Dental School, University of Campinas(UNICAMP), Piracicaba, Brazil.; 2 Department of Periodontology, Dental Research Division, Guarulhos University, Guarulhos, Brazil; 3 University of Pittsburgh, Department of Periodontics and Preventive Dentistry, School of Dental Medicine, Pittsburgh, PA, USA.; 4 Dental Science School (Faculdade de Ciências Odontológicas - FCO), Montes Claros, Brazil.

**Keywords:** titanium, dental implant, biofilm, infection, coating

## Abstract

Dental implants made of titanium (Ti) material is recognized as the leading treatment option for edentulous patients’ rehabilitation, showing a high success rate and clinical longevity. However, dental implant surface acts as a platform for microbial adhesion and accumulation once exposed to the oral cavity. Biofilm formation on implant surfaces has been considered the main etiologic factor to induce inflammatory diseases, known as peri-implant mucositis and peri-implantitis; the latter being recognized as the key reason for late dental implant failure. Different factors, such as biofilm matrix production, source of carbohydrate exposure, and cross-kingdom interactions, have encouraged increased microbial accumulation on dental implants, leading to a microbiological community shift from a healthy to a pathogenic state, increasing inflammation and favoring tissue damage. These factors combined with the spatial organization of biofilms, reduced antimicrobial susceptibility, complex microbiological composition, and the irregular topography of implants hamper biofilm control and microbial killing. In spite of the well-known etiology, there is still no consensus regarding the best clinical protocol to control microbial accumulation on dental implant surfaces and treat peri-implant disease. In this sense, different coatings and Ti surface treatments have been proposed in order to reduce microbial loads and control polymicrobial infections on implantable devices. Therefore, this critical review aims to discuss the current evidence on biofilm accumulation on dental implants and central factors related to the pathogenesis process of implant-related infections. Moreover, the potential surface modifications with anti-biofilm properties for dental implant devices is discussed to shed light on further promising strategies to control peri-implantitis.

## Resumo

Implantes dentários em titânio (Ti) são reconhecidos como principal modalidade terapêutica para a reabilitação oral de pacientes edêntulos, demonstrando uma alta taxa de sucesso e longevidade clínica. No entanto, após inserção no ambiente bucal, os implantes dentários agem como substrato para adesão e acúmulo microbiano. A formação de biofilmes em implantes dentários tem sido considerada o principal fator etiológico para induzir doenças inflamatórias conhecidas como mucosite peri-implantar e peri-implantite, sendo está última reconhecida como principal razão para falha tardia dos implantes dentários. Diferentes fatores têm sido atribuídos por promover o acúmulo microbiano em implantes dentários, levando a uma mudança microbiológica e favorecendo o dano tecidual, como a matriz do biofilme, exposição a carboidratos e interação entre reinos. Esses fatores combinados com a organização espacial de biofilmes, reduzida suscetibilidade microbiana, complexa composição microbiológica e a superfície irregular dos implantes dificultam o controle do biofilme e a morte microbiana. Apesar da etiologia bem conhecida, ainda não há consenso sobre o melhor protocolo clínico para controlar o acúmulo microbiano nas superfícies dos implantes dentários e tratar a doença peri-implantar. Nesse sentido, diferentes coberturas e tratamentos de superfície no Ti têm sido desenvolvidos objetivando a redução dos níveis microbianos e o controle das infecções polimicrobianas em implantes. Portanto, essa revisão crítica objetiva discutir a atual evidência em relação ao acúmulo de biofilmes em implantes dentários e fatores chave relacionados ao processo patogênico das infecções peri-implantares. Além disso, o potencial de alterações de superfícies com propriedades antimicrobianas para implantes dentários é discutido para ressaltar futuras estratégias promissoras no controle da peri-implantite.

## Introduction

Titanium (Ti) has a long history as a pioneering material for dental implant production, being widely used to replace missing teeth and re-stablish masticatory, occlusal, and esthetic functions ^1^ ^,^ ^2^. Although Ti-based dental implants are a well-established treatment option with a high long-term survival rate (> 92%) ^3^ ^,^ ^4^, Ti material is susceptible to the wear/corrosion (i.e. tribocorrosion) process ^5^ ^,^ ^6^ and constant microbial challenge ^7^ ^,^ ^8^ in the oral environment. Based on recent consensus reports ^9^ ^,^ ^10^, biofilm-related to dental implants is the main etiological factor to induce implant-related infections, named peri-implant mucositis and peri-implantitis. These pathologic conditions increase the risk of late implant failures ^11^, thus being a relevant problem to oral health, especially in edentulous patients ^12^. All non-surgical therapies in these cases are challenging because of the difficult access to treat the disease sites, the complex micro and macro topography of implant surface, and because of how tightly adhered these biofilms are to dental implant surfaces ^8^ ^,^ ^13^. This may explain the ineffectiveness of non-surgical treatments for peri-implantitis cases ^14^ ^,^ ^15^, even associated with systemic antibiotics ^16^. Therefore, to prevent and control microbial adhesion and accumulation on dental implant surfaces, designing antimicrobial Ti surface modifications has attracted researchers' attention visualizing the possibility of avoiding these infections, rather than treating them ^17^ ^,^ ^18^.

Besides improving peri-implant bone healing process, Ti surface modifications can promote the desired anti-biofilm effects ^19^. Antimicrobial compounds such as bioactive elements, metallic nanoparticles and ions, antibiotics, anti-infective substances, antimicrobial peptides (AMPs), and therapeutic polymers have been routinely incorporated onto Ti surfaces by numerous deposition methods ^20^. Previous studies ^21^ ^,^ ^22^ ^,^ ^23^ ^,^ ^24^ ^,^ ^25^ ^,^ ^26^ have confirmed the promising results of these emerging surfaces to control biofilm accumulation and microbial infections in animal models. However, thus far, the market lacks Ti surface modifications with trustworthy antimicrobial properties for dental implant applications in the clinical setting ^27^.

Therefore, this critical review aims to offer an updated perspective of implant-related infections and the development of Ti surface modifications with desirable antimicrobial properties for dental implant applications. In addition, we prospect this review of available scientific evidence will stimulate researchers to rationally engineer safer implant surfaces, focusing on translation to real-world applications.

### Biofilm accumulation on dental implants: a villain to be defeated

Biofilms are well-organized microbial communities enmeshed in a three-dimensional extracellular matrix ^28^ ^,^ ^29^, which create a proper environment and architecture with numerous benefits to colonizing species, such as protection against antimicrobials, improved co-aggregation, microbial metabolism, and interaction properties ^29^ ^,^ ^30^. Biofilm accumulation on biotic and abiotic surfaces is a multifaceted process mediated by the materials’ surface properties, the host response, and environmental conditions ^31^. Therefore, indigenous microorganisms from the oral microbiome living in a mutual state with the host and adhered on any surface in the oral cavity are directly affected by local or host environmental changes, which may lead to the overgrowth of pathogenic species, and often lead to the development of oral diseases ^32^. Hence, any surface inserted in the oral environment, such as dental implants, can act as an additional substrate for microbial adhesion and accumulation ^8^.

Protein adsorption on biomaterials has been pointed as the initial biological response in the human body, and this process is responsible for interceding successive cellular events, such as microbial and host cell adhesion ^33^. Interestingly, the chemical and physical properties of Ti surfaces directly affect the proteomic profile of proteins adsorbed from saliva and plasma ^34^ ^,^ ^35^. Therefore, important differences between oral surfaces and biomaterial surfaces are expected for protein adsorption and for further microbial adhesion, thus the comprehensive knowledge gained from microbial accumulation on dental surfaces cannot be simply transferred to the dental implant field before being tested experimentally.

Knowing that the oral cavity hosts nearly a thousand bacterial species detectable by high throughput 16S rRNA gene sequencing ^32^, dental implants will be exposed to a complex community of microbes once coated by the initial saliva/plasma protein layer. Thus, although teeth and implants share the same oral environment, they can show differences in their microbial ecosystems, mainly in terms of microbial species ^36^. On dental implants, *Streptococcus, Fusobacterium,* and *Capnocytophaga* species have been described as initial colonizers immediately after implant placement ^37^, whether in teeth early plaque formation involves several streptococcal species, as well as nonstreptococcal species, such as *Neisseria*, *Rothia*, and *Gemella*. ^38^ ^,^ ^39^. Thus, interactions among different species and late colonizers, recognized as the co-aggregation process, which will drive biofilm maturation, can significantly differ from dental implants and teeth, being highly site specific. Once mature, biofilms form a well-established and steady ‘climax community’ ^40^ ^,^ ^41^ known to be very stable over time ^42^.

### Implant-related infections: a prevalent and challenging problem

According to the last classification of dental implant diseases published in 2018, the current evidence shows that polymicrobial biofilms are responsible for inducing inflammatory disease processes on peri-implant sites named as peri-implant mucositis and peri-implantitis ^9^. While peri-implant mucositis is characterized by reversible inflammation in the dental implant surrounding mucosa without alveolar bone loss, peri-implantitis results from the progressive loss of supporting bone in which implants are anchored ^9^ ^,^ ^10^. Therefore, implant-related infections, mainly peri-implantitis, have been considered the main reason for dental implant treatment failure, affecting from 40% to 22% of implants by peri-implant mucositis and peri-implantitis, respectively ^43^.

Importantly, a recent adaptation from the “ecological plaque hypothesis” on tooth surfaces was used to depict a switch from a healthy to a disease state in the peri-implant mucosa and bone tissues ^19^. This biological hypothesis, based on the current evidence, describes the different factors that may trigger/contribute to disrupting the symbiotic state of implant-related biofilms, leading to an overgrowth of species with pathogenic potential, triggering inflammatory processes, or contributing to disease progression ^19^. We and others have shown that several factors may contribute to this process, such as carbohydrate exposure that favors biofilm growth matrix production, and cross-kingdom interaction between bacteria and *Candida albicans,* all that leading to subsequent environmental changes and a higher proinflammatory response ^44^ ^,^ ^45^ ^,^ ^46^. These factors, combined with the complex architecture of oral biofilms, wide microbiological composition, reduced antimicrobial susceptibility, and the irregular topography of dental implants, make it a complex disease to be controlled and eradicated ^47^. Furthermore, it may explain the non-consensus regarding the optimal clinical protocol to treat peri-implantitis and the ineffectiveness of non-surgical treatments ^15^ ^,^ ^48^. Therefore, coatings and changes on chemical/physical properties of Ti surfaces are emerging as promising approaches to improve the antibacterial properties potential of Ti-based implantable devices and to prevent implant-related infections ^27^.

### Implant surface engineering to control biofilm-related to dental implants

Ti surface modifications have been widely proposed to improve the quality of the osseointegration process and/or exert an antimicrobial property to avoid implant infections ^17^ ^,^ ^49^. Generally, implant surface modifications change the material topography by macro-, micro-, or nano-texturization with or without the incorporation of antimicrobial compounds ^19^ ^,19)^. These implant surface modifications can be performed by numerous techniques, which may be mechanical, physical, chemical, or a combination of all ^20^. In terms of antimicrobial properties, four major surface mechanisms should be considered: *i*) microbial biofilm modulation (bioactive surfaces); *ii*) microbial repelling/non-adhering properties (antifouling surfaces), *iii*) contact-killing or release-killing compounds (antimicrobial-loaded surfaces), and *iv*) ‘on-demand’ antimicrobial agent delivery (smart surfaces) ^49^ ^,^ ^50^. For the purpose of this review, Ti surface treatments were classified based on the above-mentioned antimicrobial mechanisms, as previously described elsewhere ^27^.

### Bioactive implant surfaces

The initial research focus on implant surfaces was the so-called ‘bioactive surfaces’ ^51^. In these surfaces, functional compounds [e.g., Ca, PO₄³⁻, Si, Mg, Zn, Na, N, C, Ta, Se, hydroxyapatite, bioactive glasses, growth factors, and others] were incorporated onto the Ti surface, changing the material surface properties and directly improving its biological responses ^52^. Compared to control surfaces (machined, acid etching, or grit-blasting Ti surfaces), bioactive surfaces have revealed good mechanical/tribological behavior ^53^, superior anti-corrosion performance ^54^, with bone cell stimulatory capacity ^55^, and faster *in vitro* apatite growth ^56^. Bioactive surfaces also promote higher bone matrix deposition at the implant-tissue interface, as well-documented in animal models ^57^ ^,^ ^58^. Although bioactive surfaces can be indicated to reduce the initial osseointegration period ^52^, which is clinically relevant for implant therapy with immediate/early loading protocols ^59^, these surfaces have limited anti-biofilm effect due to the absence of antimicrobial agents. Currently, the major challenge is incorporating antimicrobial elements into the Ti material able to promote a reliable and long-term antimicrobial effect without losing their bioactive capabilities overtime or reaching sub-inhibitory concentrations which can favor the emergence of hyper-resistant strains, creating a much bigger issue ^60^ ^,^ ^61^.

**Figure 1.**
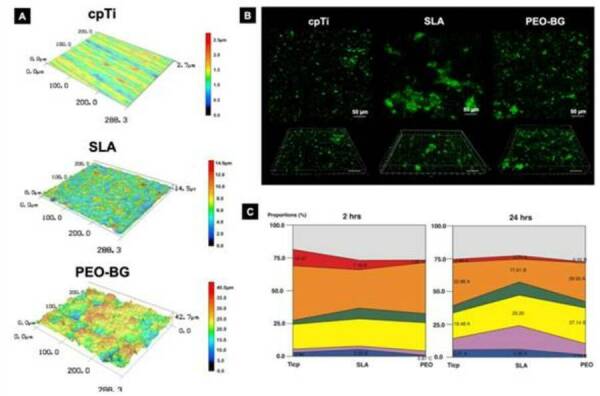
Overview of newly developed bioactive glass-based coating for implant application with microbial biofilm modulationattributes. **(A)** Representative confocal images (50× magnification) revelated the complex surface topography of the PEO-BG surface (*i.e.*, “glass grain-like”) compared to control surfaces such as machined and SLA. Surface chemistry was evaluated using energy-dispersive spectroscopy, showing stoichiometric proportions similar to 45S5 bioglass on Ti surface (data not shown). Microbiological analysis was performed after microbial adhesion (2 h) and biofilm formation (24 h) with human saliva as the microbial inoculum. Although the number of viable bacterial cells visualized by confocal imagens (live cells were stained in green by SYTO-9; scale bars 50 µm) did not differ considerably between control groups during microbial adhesion **(B)**, we untangled that the bacterial community profile adhered to the PEO-BG surface was more compatible with the resident microbiome by DNA-DNA checkboard method **(C)**. Reprinted adapted from Costa *et al.* 2020 [62]; Copyright (2020);

Regarding the antimicrobial attributes of bioactive surfaces, researchers are not only thinking about developing surfaces that could kill bacteria, but also creating a positive effect on the composition of the attached biofilm by selecting health associated bacterial species ^50^. In this context, our group recently engineered a bioactive glass-based coating by plasma electrolytic oxidation (abbreviated as PEO-BG), which was able to improve the biological responses along with promoting positive polymicrobial biofilm modulation ^62^. PEO-BG induced a microbiological shift on the implant surface colonization, promoting a decrease in diversity and a gradual depletion of pathogenic associated bacteria and an enrichment of the healthy resident microbial community (Figure 1). We showed that the surface chemistry and topography patterns of PEO-BG surface led to a shift from disease- to the health-associated microbial community, which once stable, could prevent implant-related infections ^63^. Surfaces with a biofilm modulation mechanism also seem promising to avoid the ever-increasing problem of bacterial resistance on implanted biomaterials ^64^ and are worth researching more extensively to obtain in-depth exploration for posterior use in patients.

### Antifouling implant surfaces

**Figure 2:**
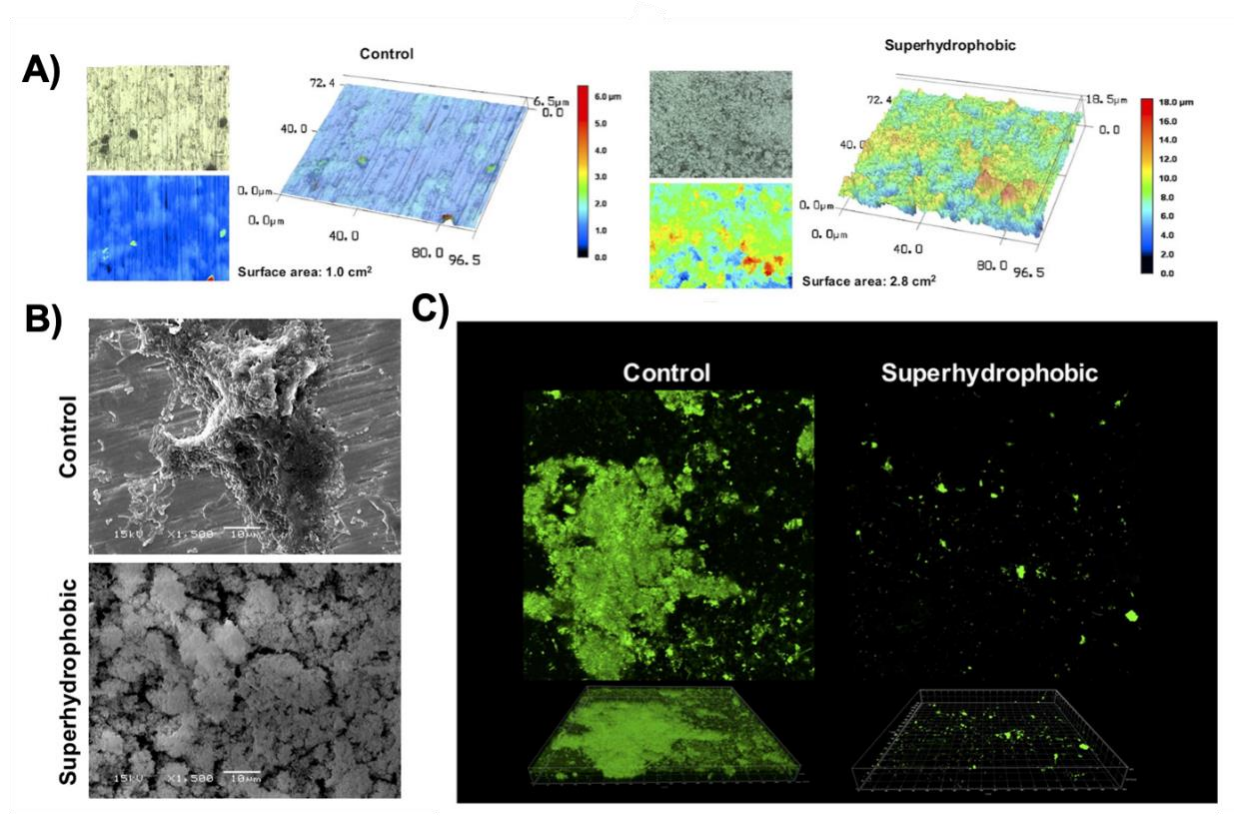
One-step superhydrophobic coating synthesized by low-pressure glow discharge plasma technology on Ti biomaterial. **(A)** Two- and three-dimensional images (150× magnification) by 3D laser scanning confocal microscopy showing the differences in the topography patterns and surface area between surfaces (non-treated control and treated). Polymicrobial biofilm was formed for 24 h using human saliva as the microbial inoculum to check the antifouling property. **(B)** Scanning electron microscopy (1,500× magnification) and **(C)** confocal images (50 μm; green - live bacteria cells stained using SYTO-9) confirmed that superhydrophobic coating dramatically reduces the microbial biofilm adhesion on Ti surface *in vitro*. Reprinted adapted from Sousa *et al.* 2020 [69]; Copyright ^2020^; with permission from American Chemical Society (License number: 5084191501822).

Since microbial adhesion is the first step of biofilm formation, designing antifouling surfaces can help to avoid implant-related infections from the beginning, creating a biofilm free surface ^65^. Implant surface properties such as wettability, surface roughness, and topography patterns play an essential role in microbial-surface interactions and directly affect bacterial adherence and colonization ^66^. For instance, increasing a surface's hydrophobicity can diminish the adhesion forces between bacteria and implant surface, thus avoiding the micro-organism ability to reach the surface and form biofilms ^65^. However, the main concern about these antifouling surfaces in the implantology field is the possible negative impact for initial protein adsorption and, importantly, for host cell adhesion processes ^67^ ^,^ ^68^. Remarkably, Souza *et al.* ^2020^ ^) (^ ^69^, developed a one-step protocol based on a low-pressure glow discharge plasma technology, to generate a superhydrophobic coating (water contact angle over 150°) for the Ti surface able to dramatically reduce the initial bacterial adhesion while allowing human fibroblast cell colonization and proliferation. This newly developed antifouling surface also allowed bacterial killing by direct contact with the oral bacteria attached to the Ti surface, tested *in vitro* and *in situ* (~ 8-fold decrease *vs*. non-treated surface) (Figure 2), reducing the load of bacterial pathogens associated with peri-implantitis. Moreover, this surface favored fibroblasts cell adhesion (at 1, 3, and 4 days of culture), probably due to the high surface roughness, as previously reviewed ^65^. Based on Wenzel and Cassel-Baxter principles ^68^, the surface hydrophobicity is determined primarily by surface roughness; therefore, this surface is an example in which the Ti surface modification led to altered physicochemical properties (highly roughened surfaces; Ra > 3.0 μm) with direct antimicrobial effect. In addition, others have shown that bactericidal effect is more pronounced on surfaces with taller structures ^70^.

In addition to superhydrophobic surfaces, nature-inspired biomimetic surfaces also have strong antifouling activities to prevent microbial accumulation ^71^. In terms of topography patterns, there is a relationship between the size scale of the bacteria and the characteristic dimensions of the surface texture to promote the anti-biofilm effect ^72^. Overall bacteria preferentially adhere to and colonize in the grooves and spaces of micro structured surfaces, but only if these recessed regions are larger than or approximately equal to their own size ^27^ ^,^ ^66^, as the size of the nanostructures increases, the bacterial adhesion forces decrease ^70^. In this sense, nanostructured Ti surfaces have been associated with antifouling and bacteriostatic effects by repelling the bacteria that cannot find sites for initial attachment ^72^. Among the nature-inspired biomimetic surfaces, the creation of nanopillars ^73^, nanocolumns ^74^, nanowires ^75^, nanospikes ^76^, spear-type ^77^, wing-like ^78^, and lotus-leaf-like ^79^ surfaces have potential to inhibited specific bacterial species (such as *Streptococcus aureus* and *Escherichia coli*) and represents a valuable strategy to be tested against oral polymicrobial biofilms related to dental implant surfaces using *in vivo* mechanistic studies.

### Antimicrobial-loaded implant surfaces

**Figure 3:**
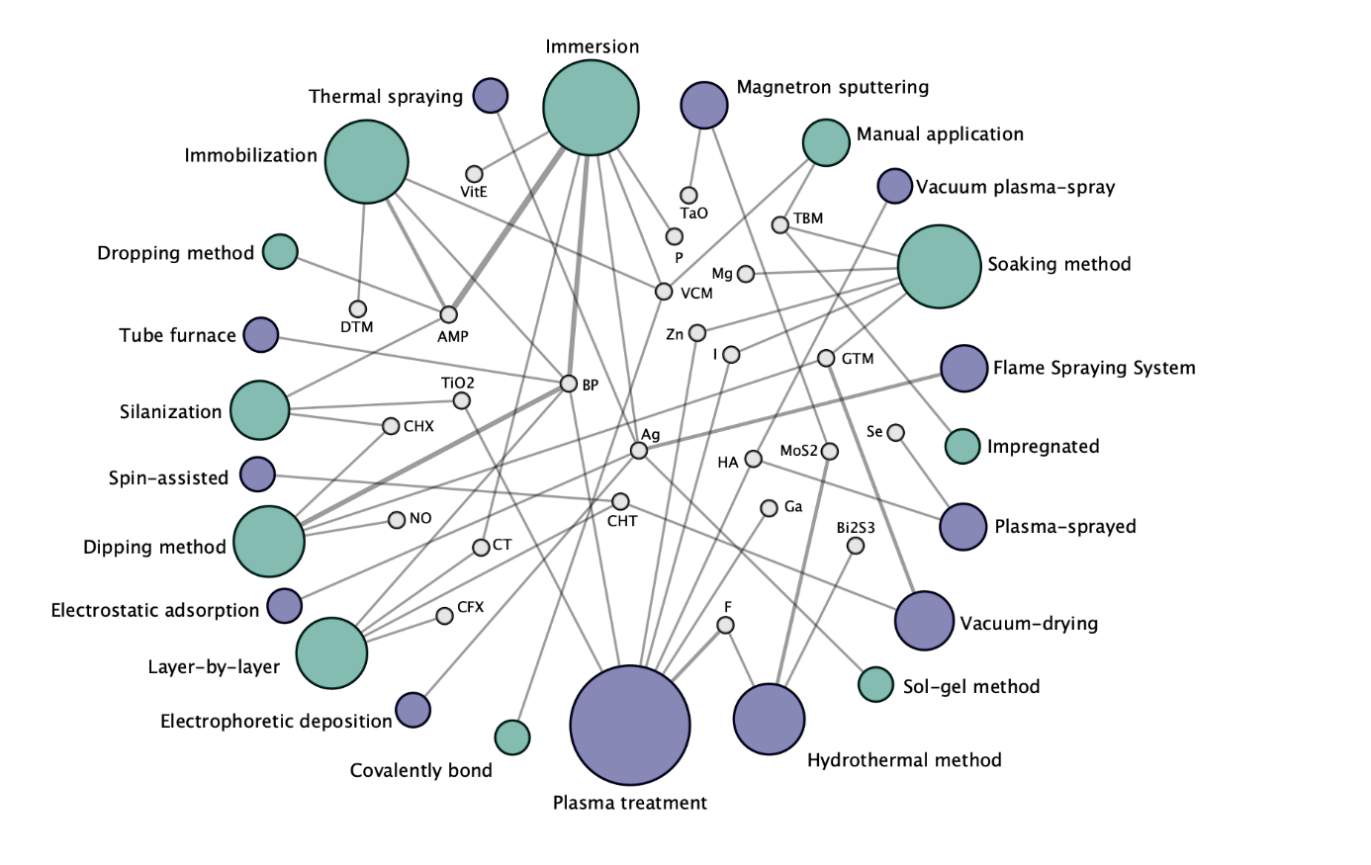
The network analysis about the most common functional compounds incorporated onto Ti surface using different deposition methods in preclinical studies. Green nodes represent the chemical deposition methods and purple nodes the physical deposition methods. The size of the node is proportional to the number of animal studies included. Gray lines represent the direct comparisons of each antimicrobial deposition method, and the line thickness is directly proportional to the number of incorporations. [ Abbreviations: AMP = antimicrobial peptides; BP = biopolymers; VitE = Vitamin E; DTM = Daptomycin; CHX = Chlorhexidine; NO = nitric oxide; CT = catechol; CFX = Ciprofloxacin; CHT = Chitosan; F = Fluoride ion; Ga = Gallium ion; HA = Hydroxyapatite; MoS2 = Molybdenum disulfide; Bi2S3 = Bismuth sulfide; Se = Sellenium ion; GTM = Gentamycin; TBM = Tobramycin; TaO = Tantalum oxide; P = Red Phosphorus; VCM = Vancomycin; Zn = Zinc ion; I = Iodine ion; Ag = Silver ion; and TiO2 = Titanium dioxide]. Reprinted from Costa *et al.* 2021 [27]; Copyright ^(^2021^)^; with permission from Elsevier (License number: 5214900523221).

Antimicrobial-loaded surfaces have been a major focus of implantology research in the last 20 years ^80^ ^,^ ^81^ ^,^ ^82^. Numerous antimicrobial compounds [e.g., metallic nanoparticles and ions, antibiotics, anti-infective substances, antimicrobial peptides (AMPs), therapeutic polymers, and others] have been employed to attempt to improve Ti surfaces antimicrobial activities ^17^. These antimicrobial surfaces can be achieved by one or multi-step process using several deposition techniques to ensure suitable bacteriostatic and bactericidal action (Figure 3) ^27^. Antimicrobial activity is achieved mainly via physical damage of the bacteria cell membrane ascribed to antimicrobial onto the surface (contact-killing) or their release in the local of infection with uptake into the bacterial cell (release-killing) ^66^. In fact, in laboratory studies, antimicrobial-loaded surfaces promote superior results in biofilm reduction than control surfaces ^83^ ^,^ ^84^. Nevertheless, the success of each implant surface is beyond the deposition process of antimicrobials.

Considering that implant-related infections are chronic conditions associated with continuous pathogenic and multi-resistant biofilm accumulation ^11^, the loaded surface must be stable in the oral conditions, present a long-term and sustained antimicrobial release that could cover both early and late-onset of infection, ideally with the possibility to be reloaded, when needed, to maintain an adequate therapeutic effect in clinical conditions ^64^. This colossal challenge can explain the reason why only a few developed antimicrobial surfaces are progressing into clinical research. Our recent systematic review ^27^ showed that only chlorhexidine ^85^, silver (Ag) ions ^86^, and titanium dioxide (TiO_2_) ^87^ antimicrobial coatings for dental implants have been tested in clinical trials, showing a limited effect on long term infection control. Thus, most of the proposed surfaces with good preclinical results are hardly scalable and could significantly raise the price of implants, with as-yet-unknown dental clinical benefits.

### Smart implant surfaces

Based on the current limitations related to the short-term effect of antimicrobial-loaded surfaces, smart surfaces engineering is growing as a novel generation of antimicrobial approaches for implant applications ^88^. Theoretically, smart surfaces are bio responsive materials that respond to 'on-demand' internal/external stimuli to start the antimicrobial agent release only when infections are present. This strategy can provide ideal antimicrobial concentrations at the precise moment and local of the infection, thus diminishing toxicity and microbial resistance. Some external (e.g., ultrasound, temperature, light, magnetic field, and electrical pulses) and internal (e.g., potential redox, enzymatic activity, O_2_ level, and pH) stimulus have been tested in biomedical applications to activate this developed smart surfaces, showing to be a reliable drug delivery strategy with reusability property to guarantee the activation of the surface several times, as needed. Furthermore, to enhance the optimization of these surfaces, dual-stimulus approaches have been recently proposed to faster both prevention and clinical treatment stages of dental ^89^ and orthopedic ^24^ infections. In a nutshell, smart implant surfaces engineering is a relatively new area and highly promising, but has yet to be studied thoroughly and systematically for dental implants therapy in the near future.

## Final remarks, challenges, and future perspectives

A significant concern emerging from current Implant Dentistry is the increased prevalence of peri-implant infections and the lack of consensus on the most effective therapeutic technique to treat such diseases. Often, diseased implants need to be removed, damaging bone and supporting tissues and creating a bigger problem to be solved prior the possibility of new implant placement ^90^. To overcome this issue, new strategies for peri-implant biofilm control are urged to be developed, targeting the long-term predictability of the implant treatment. In this regard, emerging surface modifications with antimicrobial and/or antifouling properties have gained a burst of attention, which motivated us in this review to reflect what is currently available and what are the future directions in this field.

There is no doubt that the first developed antimicrobial surfaces associated with the incorporation of bioactive elements or antimicrobial drugs have frustrated us to some extent. Such surfaces work in the principle of contact-killing or drug release ^84^ ^,^ ^91^, but their antimicrobial effect is not sustainable to match the chronic process of peri-implant infections. To worsen the scenario, up to now, there has been no success in creating a surface able to be reloaded with a fresh antimicrobial agent to replace the released one on dental implants surfaces, partially because of the difficulty of access to these sites. Importantly, the burst and uncontrolled release of drugs can have an impact on the development of bacteria-resistant strains and can load to cytotoxicity to surrounding host cells. We also face many studies using non-reliable *in vitro* and *in vivo* models that fail to mimic the oral environment, the human microbiota, and the complex nature of the disease. No wonder these surfaces have not been extensively tested in randomized controlled clinical trials nor gained the implant market, even though it might be a turning point to fight such disease.

Currently, in an interdisciplinary and multifaceted approach, researchers have dedicated their efforts to developing the so-called smart/intelligent surfaces. Those surfaces respond to certain stimuli to release the antimicrobial agent when precisely needed, in a site-specific manner. However, considering that the peri-implant disease has an immune-inflammatory nature, just controlling the biofilm is not enough and there is a need to incorporate immunomodulatory agents with anti-inflammatory properties to control the host response as well ^92^ ^,^ ^93^. This should definitely be considered when these so called smart surfaces are advanced enough, so that they can also modulate the exacerbated host defense, often responsible for tissue destruction ^92^ ^,^ ^93^. Interesting, there is emerging evidence showing an important role of the host immune modulation when implants present bone loss around them, as they can be seen as foreign bodies that elicit a foreign body response after placed in contact with bone tissue ^92^ ^,^ ^94^. Similar reactions, including chronic inflammation, can also be seen in the interface of orthopedic implants even though they are not always exposed to bacteria, leading to aseptic loosening, often linked to a reactivation of the inflammatory-immune system ^95^.

Recent evidence is accumulating and suggesting that the presence of Ti products around dental implants may contribute to microbial dysbiosis and peri-implantitis ^96^, and even in aseptic conditions (without the presence of microbial biofilm) Ti products released from the surface may result in damage to host tissues by negatively impacting on cell homeostasis, increasing inflammatory reaction in the surrounding tissues, bone loss, and implant detachment ^97^. Thus, blocking both the inflammation and osteoclastogenesis locally by means of a slow-release Ti surface treatment could be an interesting option for host modulation in order to prevent bone loss around implants ^98^. Although it has not been experimentally tested yet, this could be a promising therapeutic approach for the clinical management of peri-implant bone loss. Importantly, regardless of how these new surfaces are developed, it is important to consider their stability in the oral environment to avoid leaching of Ti particles and degradation products to be released in oral and nonoral tissues, further exacerbating the inflammatory process.

Another field of investigation with promising outcomes is the nature-inspired surfaces to provide antifouling activity under safe conditions ^71^. There is a wide variety of surfaces topographies to explore dedicated to the micro and nano world that interact with the bacteria in different ways. However, it is noticeable that those surfaces may also reflect on the human cell behavior; therefore, an equilibrium in the antifouling property is essential to be reached in order to maintain the surface’s *in vivo* compatibility. In this regard, machine-learning algorithms to engineer reliable surfaces are in-hand strategies to better explore novel surfaces architecture ^99^.

Finally, to smartly win the never-ending war against polymicrobial infections on dental implants and gain potential breakthroughs in the surface-engineering implant field is important to deeply recognize the strategies of our enemy, understand the precise mechanism of etiopathogeneses, and the risk factors of the peri-implant disease. Will we win the war? We do not know precisely, but significant knowledge is still to be explored by well-designed studies.
